# Efficacy, safety and mechanism of Simiaoyongan decoction in the treatment of carotid atherosclerotic plaque: a randomized, double-blind, placebo-controlled clinical trial protocol

**DOI:** 10.1186/s12906-024-04555-6

**Published:** 2024-07-22

**Authors:** QinHua Fan, ZhongJian Tan, WenQuan Su, QingXiao Li, Dian Jin, YaWei Du, LiPing Zhang, ShengXian Wu

**Affiliations:** Dongzhimen Hospital, Beijing University of Chinese Medicine, Beijing, China

**Keywords:** Simiaoyongan Decoction (SMYA), Atherosclerosis (As), High resolution magnetic resonance imaging (HR-MRI), Traditional Chinese medicine (TCM), Randomized controlled trial

## Abstract

**Introduction:**

Chronic inflammation is the major pathological feature of Atherosclerosis(As). Inflammation may accelerate plaque to develop, which is a key factor resulting in the thinning of the fibrous cap and the vulnerable rupture of plaque. Presently, clinical treatments are still lacking. It is necessary to find a safe and effective treatment for As inflammation. Simiaoyongan Decoction (SMYA) has potential anti-inflammatory and plaque protection effects. This protocol aims to evaluate the efficacy, safety, and mechanism of SMYA for patients with carotid atherosclerotic plaque.

**Methods/design:**

The assessment of SMYA clinical trial is designed as a randomized, double-blind, placebo-controlled study. The sample size is 86 cases in total, with 43 participants in the intervention group and the control group respectively. The intervention group takes SMYA, while the control group takes SMYA placebo. The medication lasts for 14 days every 10 weeks, with a total of 50 weeks. We will use carotid artery high resolution magnetic resonance imaging (HR-MRI) to measure plaque. The plaque minimum fiber cap thickness (PMFCT) is adopted as the primary outcome. The secondary outcomes include plaque fiber cap volume, volume percentage of fiber cap, lipid-rich necrotic core (LRNC) volume, volume percentage of LRNC, internal bleeding volume of plaque, internal bleeding volume percentage of plaque, plaque calcification volume, volume percentage of plaque calcification, lumen stenosis rate, average and a maximum of vessel wall thickness, vessel wall volume, total vessel wall load, carotid atherosclerosis score, hs-CRP, IL-1β and IL-6, the level of lipid profiles and blood glucose, blood pressure, and body weight.

**Discussion:**

We anticipate that patients with As plaque will be improved from SMYA by inhibiting inflammation to enhance plaque stability. This study analyzes plaque by using HR-MRI to evaluate the clinical efficacy and safety of SMYA. Moreover, we conduct transcriptome analysis, proteomic analysis, and metagenomic analysis of blood and stool of participants to study the mechanism of SMYA against As plaque. This is the first prospective TCM trial to observe and treat As plaque by inhibiting inflammatory reaction directly. If successful, the finding will be valuable in the treatment of As plaque and drug development, especially in the “statin era”.

**Trial registration number:**

This trial is registered on Chinese Clinical Trials.gov with number ChiCTR2000039062 on October 15, 2020 (http://www.chictr.org.cn).

## Introduction

Atherosclerosis (As), occurs in the middle and large arteries, chronic inflammation is its major pathological feature, which may give rise to cardiovascular diseases (CVD), such as ischemic heart disease (IHD) and apoplexy [1]. Although there are excellent lipid-lowering treatments, As-related diseases remain the key source of disability and death worldwide [2]. In 2019, the number of CVD patients reached 523 million, and the death toll was about 18.6 million [3]. It is reported that by 2030, the number of CVD deaths will reach 23.6 million [4]. As-related diseases have high morbidity and mortality, medical expenses are expensive, and impose a huge economic burden on society and families [5, 6]. In America, the cost related to IHD was evaluated to rise from $126.2 billion in 2010 to $177.5 billion in 2040, with a 41% increase in cost [7]. About 80% of worldwide CVD deaths happen in low- and middle-income countries, in these countries, the burden of CVD continues to rise because of the transformation of epidemiology [6]. Early detection of As is mostly in peripheral arteries and carotid arteries (CAs) [8]. CAs seem to be the most significant site of As in the clinic, besides coronary arteries. According to statistics, up to 20% of ischemic stroke patients were caused by carotid As [9]. In 2020, the global carotid plaque patients were evaluated to be 21.1% in the population aged 30–79, equivalent to 816 million affected people [10]. In the global trend of the aging population growing background, more people will be affected by carotid As in the future.

Chronic non-resolving inflammation is an important feature of As [11]. Conventional therapy focuses on lowering blood fat, although plenty of patients have reached goal blood fat levels, they still suffer recurrent clinical events. Under inflammatory conditions, lipid accumulation, and immune cells continuous recruitment triggers the death of lesional cells, resulting in the expansion of the lipid-rich necrotic core (LRNC). Plaque necrosis, accompanied by thinning and fragile fibrous cap, may lead to plaque rupture resulting in acute luminal thrombosis, which will occur in clinical acute events, such as myocardial infarction (MI) or stroke [12]. As a result of the coexistence of inflammatory plaque and coronary artery system, the risk of repeated CVD in patients after MI is 10–12% within one year and 18–20% at three years [13]. The increased residual risk of cardiovascular events is mainly due to inflammation, anti-inflammatory treatment has become a major study direction in the prevention and treatment of As. Therefore, new anti-atherosclerotic therapies, particularly those that can better control inflammation reactions, are needed. At present, As anti-inflammatory medicine is on the rise, such as targeted therapeutic agents which are targeting inflammatory factors, Canakinumab and Tocilizumab, and classic anti-inflammatory medicines, colchicine, and methotrexate. LoDoCo2 and COLCOT trials have proved that anti-inflammatory treatment indeed improves As [14–16]. Yet these potential agents are emphasized may cause a raise in cancer risk and damage to the defense system, and have side effects such as reducing immunity and increasing the risk of infection [11]. Safe and effective treatment for As inflammation is lacking all along.

In recent years, Traditional Chinese Medicine (TCM) has had a wide-ranging impact and is recognized in many regions and countries [17]. Simiaoyongan Decoction (SMYA) is a traditional Chinese formula for treating vascular inflammation. SMYA consists of four traditional Chinese herbs, the composition shown in Table 1, has the effect of clearing heat, detoxifying, invigorating the circulation of blood, and unblocking circulation tracts. SMYA has a significant effect on various diseases, which are based on As lesions, for instance, coronary atherosclerotic heart disease [18, 19]. *Lonicera japonica* Thunb has effects on antibacterial, antiviral, anti-inflammatory, antioxidant, and others, and also has certain effects on the immune system and cardiovascular systems [20]. Chemical composition studies showed that SMYA contained organic acids, flavonoids, iridoids, phenylpropanoids, volatile oil, triterpenoid saponins, polysaccharides, and so on [21–24]. Some experimental research has proved that SMYA may significantly reduce the level of cardiovascular inflammation index hs-CRP and the content of its upstream inflammatory factors, IL-6, IL-1β and so on, the effect mechanism probably related to TLR4, NF-κB, Dll4 or other relevant signal transduction pathways [25–27]. In As rabbit experiment, it was found that the level of some inflammatory cytokines in blood and plaque decreased after taking SMYA, meanwhile, the content of LDL-C and TG also reduced, the thickness of fiber cap and the content of smooth muscle cells (SMCs) might increase, and these improvements contributed to stabilizing vulnerable plaque (VP) [28]. Our team’s clinical research indicated that taking SMYA continuously for 2 weeks might decrease the average serum hs-CRP of patients with carotid atherosclerotic plaque by 57.65%, its curative effect was fast, the repeatability was strong, and the safety was good [29]. 

**Table 1:** Composition of Simiaoyongan Decoction

Ingredients	Latinname	Family	Content
Jinyinhua	*Lonicera japonica Thunb*	*Caprifoliaceae*	90 g
Xuanshen	*Scrophularia ningpoensis Hemsl*	*Scrophulariaceae*	90 g
Danggui	*Angelica sinensis (Oliv.) Diels*	*Apiaceae*	60 g
Shenggancao	*Glycyrrhiza uralensis Fisch*	*Fabaceae*	30 g

Yet, the previous atherosclerosis-related TCM research mainly focused on experiments and mechanism exploration, clinical studies were few. Meantime, in terms of efficacy indicators, most clinical studies were limited to assessing the effect of TCM on inflammatory factors and blood lipids in serum or using primary or subjective means such as ultrasound to evaluate As plaque. High-quality randomized, double-blind, and controlled studies on the treatment of As in TCM are lacking.

Numerous magnetic resonance imaging (MRI) studies have identified characteristics of carotid plaque related to cerebral vascular diseases [30], but there is no clinical research associated with TCM. Thus, we design this randomized, double-blind, parallel-controlled study and high resolution magnetic resonance imaging (HR-MRI) is used to correctly assess the efficacy of SMYA in the treatment of carotid atherosclerotic plaque. In the meantime, this study will also explore the internal mechanism of SMYA in the treatment of As, through transcriptome analysis, proteomic analysis, and metagenomic analysis of blood and stool. If successful, the finding will be valuable in the treatment of As plaque and drug development, and provide a reliable evidence chain.

## Methods

### Study design

The assessment of SMYA clinical trial is designed as a randomized, double-blind, placebo-controlled study. The sample size is 86 cases in total, with 43 participants in the intervention group and the control group respectively. The protocol version number is V2.0, version date is September 29th, 2020. The study has also been registered at http://www.chictr.org.cn(ChiCTR2000039062, October 15, 2020). This trial has the following objectives: (1) To evaluate the efficacy of SMYA for carotid atherosclerotic plaque by HR-MRI. (2) To estimate the safety of SMYA. The vital signs, adverse events (AEs), routine blood tests, liver function tests, and kidney function tests are the main indicators to evaluate safety. (3) To research the internal mechanism of SMYA for carotid atherosclerotic plaque by blood transcriptome analysis, blood proteomic analysis, and stool metagenomic analysis. The protocol will be described according to SPIRIT-TCM Extension 2018 (Additional file 1) [31]. 

### Recruitment

**Fig. 1:**
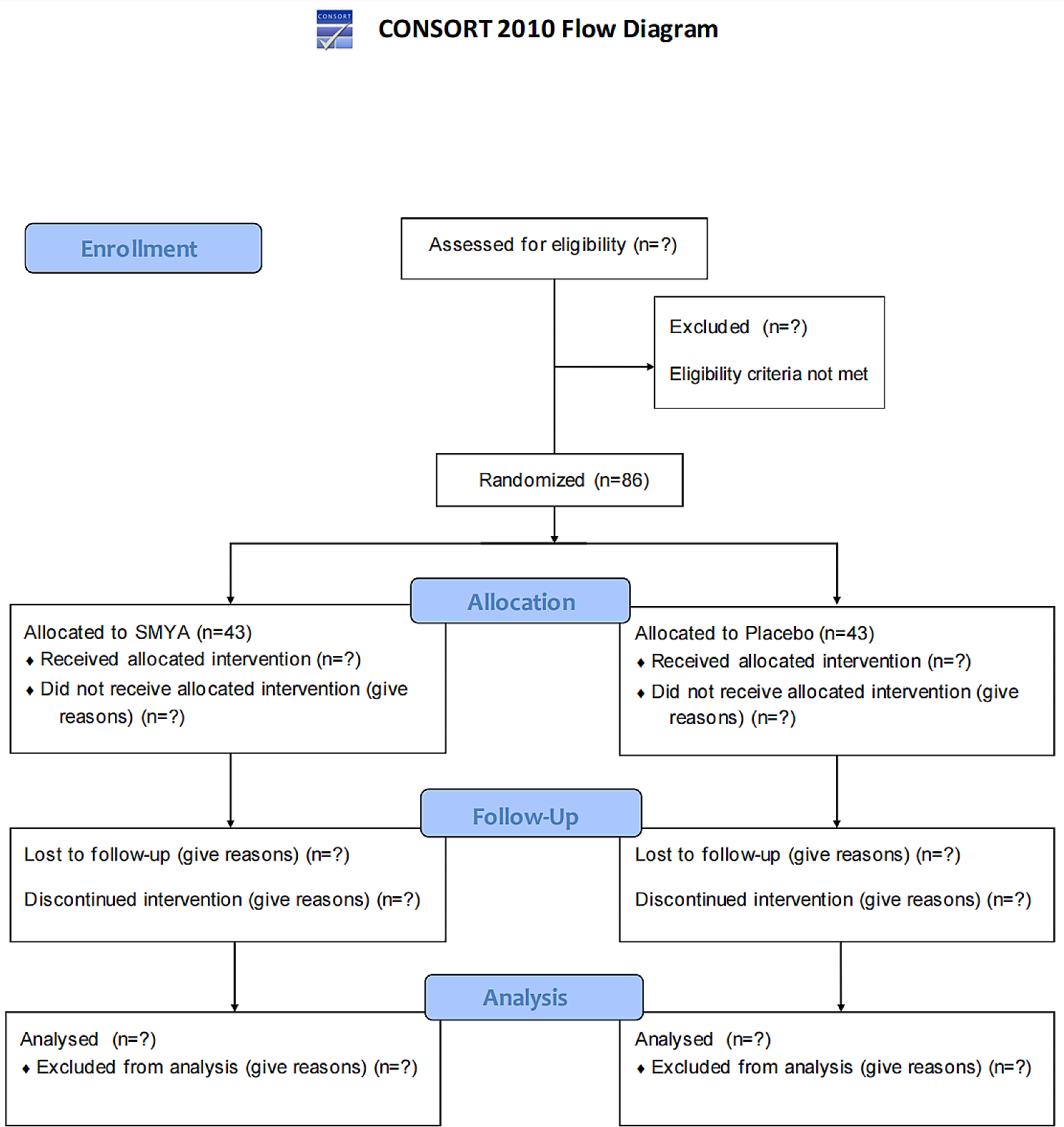
Participant flowchart

The study will be conducted in Dongzhimen Hospital Affiliated to Beijing University of Chinese Medicine (DHABUCM) (Beijing, China). We will release recruitment information through posters, email push, social media publicity, and outpatient service. Patients are involved in the conduct of our research. All subjects must sign an informed consent form before enrollment. Figure 1 shows the flow chart of the trial. At the time of manuscript submission, patient recruitment for the trial is ongoing. The expected recruitment rate is 2 patients per week. The first patient was included on October 20, 2022, and the study is expected to be completed in December 2025.

### Participants selection

#### Diagnostic criteria

The observed vascular segments are bilateral common CA and internal CA. Color Doppler ultrasound indicates that the local bulge in the target vascular segment is > 0.5 mm from the arterial lumen or exceeds 50% of the surrounding intima-media thickness (IMT) value, or IMT > 1.5 mm, diagnoses carotid atherosclerotic plaque [32, 33].

#### Inclusion criteria

1. Participants with carotid atherosclerotic plaque under the diagnosis of Color Doppler ultrasound.
2. hs-CRP ≥ 2 mg/L.
3. Participants aged 40–80 years, male or female.
4. Participants can stick to taking medicine for a long time.
5. Signing informed consent voluntarily.

#### Exclusion criteria

1. Participants with acute or subacute bleeding in plaque.
2. Participants with complete calcification and fibrosis of plaque.
3. Participants contraindicated by MRI, such as carrying cardiac pacemaker, artificial tooth, cardiac stent, and so on.
4. Participants with active ulcers and bleeding tendency.
5. Participants take anticoagulant medicine (such as warfarin) for a long time or are taking dual antiplatelet medicine.
6. Participants with severe arrhythmia, atrial fibrillation, and heart failure, and other severe diseases including the liver, kidney, hematopoietic system, endocrine system, and respiratory system.
7. Alanine aminotransferase (ALT) and/or aspartate aminotransferase (AST) ≥ 1.5 times the upper limit of normal, and/or creatinine (Cr) higher than normal.
8. Participants with any other life-threatening or serious diseases and are unable to complete 50 weeks of treatment.
9. Pregnant or lactating women, and women who have recent pregnancy plans.
10. Allergic to components of the product.
11. Participants with other diseases or mental disorders, who are deemed may limit efficacy evaluation or patient follow-up by the researcher.
12. Participants who have entered into clinical trials of other medicines within 4 weeks.

#### Drop-Out criter

1. Participants cannot tolerate treatment medicines or control medicines during the trial.
2. Participants with some serious complications, concomitant diseases, or special physiological changes occurring during the trial, and are not suitable to continue the study.
3. During the trial, participants who have poor compliance, used medicines less than 80% of the requisite amount or more than 120% of the requisite amount.
4. Participants added medicines prohibited under this protocol.

#### Voluntary withdrawal of participants

1. For whatever reason, the subjects are reluctant or unable to keep the clinical trial.
2. Although subjects are not explicitly proposed to withdraw from the trial, they are no longer subject to treatment and examination and lost to follow-up.

#### Conditions for suspension

1. Any serious adverse event (SAE) takes place during the treatment period.
2. Any gross fault is found in the clinical trial plan, which leads to difficulty in estimating treatment efficacy.
3. Any significant deviation in the implementation of the protocol, if continues, it will be difficult to estimate treatment efficacy.
4. The principal investigator requests to discontinue the trial.
5. The drug supervision and administration department request to suspend the trial.

### Sample size calculation

According to our previous studies, on the basis of maintaining the original lipid-lowering therapy, in contrast with placebo, SMYA is expected to increase the plaque minimum fiber cap thickness (PMFCT) by an average of 40% µm. The standard deviation of the experimental group in the intervention of carotid plaque FCT is about 38, and the control group is about 36. This trial is designed as a 1:1 parallel control, α = 0.01, power = 90%, two-sided test, and the SAS (6.12) is adopted to evaluate the sample size. According to the calculation, 36 participants are needed for each group. After considering drop-out rates, the sample size of this protocol is 43 participants in the intervention group and the control group respectively, with a total of 86 cases.

### Randomization, allocation and blinding

The stratified blocked randomization method will be used in this protocol. A random code list will be generated by SAS (6.12), and The intervention group and the control group will be randomly assigned to the enrolled participants in a ratio of 1:1. This study selects double-blind method, subjects, clinical researchers, drug managers, statisticians, and other staff members will be blinded. The trial is also designed as a two-stage blind. The blinding is conducted by irrelevant personnel, and the blind record is formed. In order to ensure the quality of the blind method, all medicaments, including placebo, are required to be packaged uniformly. According to the *Standard for the Use of Food Additives in the National Standard for Food Safety* [34], the placebo is made from 5% raw materials and 95% dextrin based on the type and volume of the raw materials. Adding an extremely low dose of the raw material makes this placebo similar in appearance, color, smell, and taste to the therapeutic drugs, but without any pharmacological effects [35, 36].

The blind bottom will be sealed and stored. If the blind bottom is opened, it is necessary to indicate the person who opened it, the date, the reason, etc., and at the same time record it in the case report form (CRF). In case of emergency, or when the patient needs to be rescued and necessary to know what medicines the patient is taking, The corresponding emergency letters must be opened and read in the presence of researchers and research leaders. Once the emergency letter is opened, the participant with this number will withdraw from the trial, at the same time, CRF will record all details. All emergency letters shall be taken back with the CRFs after the study for blind review.

### Intervention method

The intervention group takes SMYA, while the control group receives SMYA placebo (containing 95% dextrin and 5% SMYA). Both groups of drugs are taken orally, 1 bag/time, 2 times/day. The medication lasts for 14 days every 10 weeks, with a total of 50 weeks. The investigational drugs and placebo are granules, provided by Beijing Tcmages Pharmaceutical Co., Ltd (Beijing, China) according to the double-blind principle. The placebo is the same as treatment medicine in color, smell, taste, and packaging. Table 2 shows the schedule of enrollment, interventions, and assessments.

**Table 2:** Schedule of enrollment, interventions and assessments

Stage procedure	Screening stage/Baseline	During treatment								After treatment
	-1 ~ 0days	2weeks	12weeks	14weeks	24weeks	26weeks	36weeks	38weeks	48weeks	50weeks
Visit	1	2	3	4	5	6	7	8	9	10
Patient screening	×									
Sign informed consent form	×									
Fill in demographic information	×									
Past medical history	×									
Medical comorbidities and current medication	×									
Physical examination	×	×	×	×	×	×	×	×	×	×
General examination	×	×	×	×	×	×	×	×	×	×
Carotid artery ultrasound	×									×
Carotid artery HR-MRI	×									×
Inflammatory cytokines tests	×	×	×	×	×	×	×	×	×	×
Blood fat and blood sugar	×	×	×	×	×	×	×	×	×	×
Weight	×	×	×	×	×	×	×	×	×	×
Blood pressure	×	×	×	×	×	×	×	×	×	×
Liver function tests and kidney function tests	×	×	×	×	×	×	×	×	×	×
Routine blood investigations	×	×	×	×	×	×	×	×	×	×
Blood transcriptome analysis	×	×	×	×	×	×	×	×	×	×
Blood proteomic analysis	×	×	×	×	×	×	×	×	×	×
Stool metagenomic analysis	×	×	×	×	×	×	×	×	×	×
Adverse events record	×	×	×	×	×	×	×	×	×	×
Combination therapy medication record		×	×	×	×	×	×	×	×	×
Distribution of the medication	×		×		×		×		×	
Summary of the remaining medication		×		×		×		×		×
Trial Summary										×

#### Guidelines for drug combination

During the trial, original lipid-lowering medicines are allowed to be used, but Western and Chinese medicines that may lower serum hs-CRP or other inflammatory factors are not allowed, for example, antibiotics, Canakinumab, and colchicine. If the participants have concomitant diseases, which they must continue to maintain these medicines or treatments, the diseases, treatment, dosage, usage and use time shall be recorded in the CRF, in order to analyze and report when summarizing. At each visit, the researcher detailed records of whether participants took medicine on time, and recovered the participants’ remaining medicines to judge treatment compliance. Patient compliance = Actual total medication/Total medication required by the protocol×100%.

#### Outcome evaluation

##### Primary outcome

PMFCT should be recorded at baseline (visit 1) and after treatment (visit 10), and evaluated at the study endpoint.

**Secondary outcome**

1. Plaque fiber cap volume, Volume percentage of fiber cap, LRNC volume, Volume percentage of LRNC, Internal bleeding volume of plaque, Internal bleeding volume percentage of plaque, Plaque calcification volume, Volume percentage of plaque calcification, Lumen stenosis rate, Average and maximum of vessel wall thickness, Vessel wall volume, Total vessel wall load (vessel wall volume/blood vessel total volume) will be measured at baseline (visit 1) and at the end of the treatment (visit 10), evaluated at the study endpoint.
2. Carotid atherosclerosis score (CAS) [37] will be recorded at baseline (visit 1) and at the end of the treatment (visit 10), evaluated at the study endpoint.

i. Low risk: When MWT ≤ 2 mm, CAS = 1.
ii. Medium and low risk: When MWT > 2 mm and LRNC ≤ 20%, CAS = 2.
iii. Medium and high risk: When MWT > 2 mm and 20%< LRNC ≤ 40%, CAS = 3.
iv. High risk: When MWT > 2 mm and 40%< LRNC, CAS = 4.
  (3) hs-CRP, IL-1β, and IL-6 will be recorded at baseline (visit 1), during the treatment period (visit 2, 3, 4, 5, 6, 7, 8, 9 ) and at the end of the treatment (visit 10), evaluated at the study endpoint.
  (4) Blood lipid (TG, TC, HDL-C, LDL-C) and blood glucose will be recorded at baseline (visit 1), during the treatment period (visit 2, 3, 4, 5, 6, 7, 8, 9 ) and at the end of the treatment (visit 10), evaluated at the study endpoint.
  (5) Blood pressure and weight will be recorded at baseline (visit 1), during the treatment period (visit 2, 3, 4, 5, 6, 7, 8, 9 ), and at the end of the treatment (visit 10), evaluated at the study endpoint.

### Safety outcome

General physical examination, including temperature, respiration, heart rate, and routine blood tests, liver function tests (ALT, AST), kidney function tests (Bun, Cr) are recorded at baseline (visit 1), during the treatment period (visit 2, 3, 4, 5, 6, 7, 8, 9) and at the end of the treatment (visit 10). In the meantime, possible AEs will be observed at any time after administration and will be recorded at any time. Subjects who are abnormal after treatment but normal before treatment will be monitored periodically until they return to normal. All AEs will be followed up until the participants are properly resolved.

### Mechanism investigation

To clarify the mechanism of SMYA in treating patients with carotid atherosclerotic plaque, stool, and blood are collected at baseline (visit 1), during the treatment period (visit 2, 3, 4, 5, 6, 7, 8, 9 ), and at the end of the treatment (visit 10), followed by transcriptome analysis, proteomic analysis, and metagenomic analysis. The fecal collection process will be carried out following the “Standards for Collection, Preservation, and Transportation of Fecal Samples in TCM Clinical Trials” [38]. 

### Quality control

#### Quality control of HR-MRI

Use 3.0T HR-MRI to scan the target vascular segment. By injecting Injection Dimeglumine Gadopentetate intravenously (according to body weight, 0.2 ml/kg or 0.1mmol/kg per time, the maximum dosage is 0.4 ml/kg at a time per time, Beijing Beilu Pharmaceutical Co., LTD ) to enhance T1-weighted imaging, we will measure the PMFCT and other plaque components. All carotid HR-MRI examinations are carried out in DHABUCM. We will use the CA coil to assist imaging and help to fix the participants’ necks and maintain a unified posture. The inspections require the same equipment and the same doctor to operate and third-party blind ultrasound image analysis is performed. All research information, including research institutions, participants, and technicians, is digitally shielded in the image. The images will be sent to Plaque Vision (Beijing) Diagnostic Technology Co., LTD., and be analyzed by two experienced clinicians using “MRI-PlaqueView” independently.

#### Quality control of trial

Before the clinical trial, all researchers and relevant staff, such as clinical researchers and drug administrators will receive unified training which is associated with a trial-specific process. The clinical researchers should fill in all cases according to the requirements of CRF, and as the original record, it cannot be changed. Laboratory data and imaging data in trials will be recorded, in addition, the original report (or copy) should be pasted on CRF. The data that are significantly higher or beyond the clinically acceptable range should be verified, and the doctors participating in this trial should make necessary explanations. The clinical supervisors regularly monitor and visit, sample and check the original data, hold regular meetings on specific issues, and timely feedback information in the form of monitoring reports and project briefings to improve the monitoring quality.

Investigators will fill in the CRFs truthfully and inspectors will check data regularly. The original record shall be kept clear and visible when modifying, and the correction shall be signed and dated by the investigators. CRFs will be submitted to the clinical trial data administrators after being checked by the supervisors. The transmission among researchers, supervisors, and data administrators will have special records. Data administrators will check data again before entering data, and any questions and answers will be saved as question forms for future reference.

### Data management

In drug clinical trial institutions of DHABUCM, EpiData3.1 data management software is used, and establish a data bank. Inputers use double entries independently to input data. After data entry, spot-check some CRFs to define the entry quality and deal with existing problems. The data administrators check the data bank with the main researchers. The original CRFs shall be filed and saved after data entry and verification as required. Electronic data files containing data banks, inspection procedures, analysis results, codebooks, and description files, have several backups and will be saved on different record media properly. All original files should be saved according to “China’s Drug Clinical Trial Quality Management Standard” within the specified time.

### Statistical analysis plan

This study will adopt an intention-to-treat analysis. And statistical analysis will be conducted by special statisticians in a blind way. SAS (6.12) will be used for statistical analysis, all statistical tests are conducted by two-sided tests, and *p* ≤ 0.05 is regarded as statistically significant. We will first analyze the baseline balance to explain whether the intervention group and control group of basic conditions are comparable. Quantitative metrics will be described with a number of cases, mean, standard deviation, median, minimum, and maximum, and t test or Wilcoxon rank sum test will be adopted to compare two groups. Qualitative metrics will be listed in frequency and percentages, and chi-square test or Fisher’s exact test will be conducted to compare two groups. For the analysis of combined medication, we will list the number and percentage, and analyze the differences between the two groups by the chi-square test or Fisher’s exact test. Analysis of treatment compliance will list the number and percentage of cases with experimental drugs < 80%, > 120%, 80-120%, and chi-square test or Fisher’s exact test will be conducted. In terms of safety analysis, we will describe the incidence of AEs and SAEs during the treatment period, and list the detailed tables of AEs and SAEs.

### Subgroup analyses

We will conduct subgroup analysis on the primary outcome and secondary outcome according to whether participants are receiving lipid-lowering therapy.

## Discussion

Maintaining plaque stability is of great significance in inhibiting the progress of As and preventing CVD. High proportion of LRNC and thin fibrous cap are the main pathological features of VP. Fiber cap is caused by accumulation from an inflammatory cell-rich fatty streak damage [39, 40]. Erosion and fracture of the fiber cap may bring about ruptured plaque, causing acute fatal thrombosis eventually [41]. The rupture of VP is the direct cause of 60-80% of acute cardiovascular events [42]. In unstable patients, for instance, acute coronary syndrome (ACS), nearly all main coronary circulations are widely lesion showing multiple VPs, during ACS, the pan-coronary vulnerability can potentially derive from an arterial inflammation that is widespread [43]. A large number of inflammatory cytokines and proteases such as matrix metalloproteinases, macrophages, CRP, and IL-1 are found in ruptured plaque [44, 45], inflammation is an important factor leading to plaque vulnerability. These inflammatory factors and proteases may inhibit SMC proliferation, induce SMC and endothelial cell apoptosis, participate in endothelial cell denudation, inhibit collagen synthesis by SMC, and degrade collagen fibers, erode plaque fiber cap [43, 46]. Collagen synthesis decreases, but degradation increases, fiber cap becomes thin and fragile, which may aggravate the vulnerability and rupture of plaque. The development of inflammation theory has turned the goal of treating As to maintain plaque stability. Low-level host inflammation may be a crucial characteristic of unstable patients [43], reduce the level of inflammatory factors such as hs-CRP and reduce As inflammatory reactions may help to strengthen plaque stability. Targeted therapeutic drugs, for example, Canakinumab and Ziltivekimab, are expensive and have serious side effects, while cheap methotrexate is unable to effectively regulate arterial inflammation [47]. Under the background of clinical treatment deficiency, it is imperative to find a safe and effective treatment for plaque inflammation of As.

SMYA is a compound prescription of TCM. The four Chinese herbal medicines in it have potential effects on inhibiting inflammation, stabilizing plaque, and treating As. *Lonicera japonica* Thunb is the most important herb in SMYA formula, and can clearly inhibit the levels of hs-CRP in patients with carotid artery plaques [29]. Experimental studies have shown that the extract of *Scrophularia ningpoensis* Hemsl may remarkably reduce the level of inflammatory factors, such as TNF-α, IL-1β, and IL-6, in As model rats, regulating the excessive expression of NF-кB in arteries [48]. The active constituent of *Scrophularia ningpoensis* Hemsl, Scropolioside B, can decrease the expression of downstream IL-1β and other pro-inflammatory factors through NLRP3 inflammasome and has anti-inflammatory effects [49]. Coumarins, contained in *Angelica sinensis* (Oliv.) Diels, can lessen the content of NO, TNF-α, COX-2, and other inflammatory factors [50]. In As animal model, it was also found that the volatile oil of *Angelica sinensis* (Oliv.) Diels could improve the steatosis of liver cells, the damage of thoracic aorta intima and myocardial fibrosis, control vascular inflammatory reaction, and delay the formation of As plaque [51]. *Glycyrrhiza uralensis* Fisch has an effect on anti-inflammatory, immune regulation, and anti-tumor [52, 53]. The active ingredient contained in it may regulate NF-κB signal pathway and related inflammatory factors. For example, Hasan SK’s study indicated that 18-beta glycyrrhetinic acid inhibited the level of COX-2 and iNOS by down-regulating NF-κB [54]. Network pharmacology showed that the effective components of *Glycyrrhiza uralensis* Fisch could regulate cell proliferation and signal transduction of apoptotic cells through multiple signal pathways, such as PI3K-AKT, MAPK, and NOD, furthermore, regulate the release of inflammatory factors, thus affecting the As process [55]. Our previous research indicated that in ApoE-/- mice SMYA could prevent the degradation of collagen in plaque, increase the area of collagen, prevent pathological remodeling of blood vessels to a certain extent, and finally stabilize plaque [56]. Experimental research in vitro and in vivo has proved that SMYA might resist inflammation and delay the development of plaque, and have a potential therapeutic effect on As.

The recent development of HR-MRI techniques has made it possible to directly evaluate plaque component and stability [57–59]. Carotid HR-MRI is generally used to estimate the vascular wall and plaque structure. However, by the MR-VPD examination of combining contrast material enhanced T1-weighted imaging and carotid HR-MRI, we can correctly quantify the plaque structure as well, containing the LRNC and fibrous cap [60, 61]. To this day, MR-VPD examination is the only clinical vascular plaque analysis technology certified by the U. S. Food and Drug Administration (FDA) and the China FDA, combined with MRI-PlaqueView software can directly evaluate the vulnerability of plaque, is the best measure of internal quantitative characterization of plaque. [62, 63] Xia et al. study compared HR-MRI imaging of plaque with histopathology and indicated that the quantitative evaluation of LRNC in carotid plaque by MRI was highly close [64]. HR-MRI is safe and non-invasive, and can help effectively monitor plaque progression and carotid risk, besides, vascular imaging has high repeatability, which may greatly improve the prediction of stroke risk [65]. HR-MRI has become a reliable imaging method for monitoring the treatment of carotid plaque and has been used in many clinical trials [66, 67]. In this study, we will use MR-VPD of carotid HR-MRI to identify the PFC, LRNC, and other plaque components in participants to ensure the accuracy and objectivity of the trial results.

TCM has been used for more than 2000 years, and traditional prescriptions have received more and more attention [68]. The research about As anti-inflammatory treatment in China started relatively late, the high-quality research on TCM is still few, and the data of large-scale trials are lacking. So far, this is the first prospective double-blind TCM clinical trial to observe and treat As plaque by inhibiting inflammatory reaction directly. Simultaneously, the follow-up time is up to 50 weeks, which may helpfully observe the therapeutic effect of TCM. On the one hand, this trial assesses the efficacy of SMYA, on the other hand, carries out transcriptome analysis, proteomic analysis, and metagenomic analysis of blood and stool of subjects to explore the internal mechanism of SMYA that impacts As plaque.

In the “statin era”, targeting decreasing the inflammation burden is a necessary way to further reduce acute cardio-cerebrovascular events . Nevertheless, the cost of developing new specific anti-inflammatory medicaments is substantial, which is also a main obstacle to converting preclinical research into commercial As treatment [13]. Consequently, we have to effort to consider repurposing existing medicines with anti-inflammatory effects to solve the residual inflammatory risk cost-effectively. The use of TCM brings more possibilities for the treatment of As inflammation. We supposed that patients with As plaque would be improved from SMYA by inhibiting inflammation to enhance plaque stability, if successful, the finding will be greatly valuable in the treatment of As plaque and drug development.

### Trial status

This study is ongoing. The first patient was included on October 20, 2022, and the study is expected to be completed in December 2025.

## Data Availability

The raw data supporting the conclusions of this article will be made available by the authors SXW, without undue reservation.

## Abbreviations

As: atherosclerosis
SMYA: Simiaoyongan Decoction
HR-MRI: high resolution magnetic resonance imaging
PMFCT: plaque minimum fiber cap thickness
CVD: cardiovascular diseases
IHD: ischemic heart disease
LRNC: lipid-rich necrotic core
MI: myocardial infarction
TCM: Traditional Chinese Medicine
CA: carotid artery
hs-CRP: hypersensitive C-reactive protein
NF-κB: nuclear factor kappa-B
TLR4: toll like receptor 4
Dll4: delta-like 4
IL-1β: interleukin-1β
TNF-α: tumor necrosis factor-α
COX-2: cyclooxygenase-2
LDL-C: low-density lipoprotein-cholesterol
HDL-L: high density lipoprotein-cholesterol
TG: triglyceride
TC: total cholesterol
SMC: smooth muscle cell
VP: vulnerable plaque
DHABUCM: Dongzhimen Hospital Affiliated to Beijing University of Chinese Medicine
AE: adverse event
ALT: alanine aminotransferase
AST: aspartate aminotransferase
BUN: blood urea nitrogen
Cr: creatinine
IMT: intima-media thickness
SAE: serious adverse event
CRF: case report form
CAS: Carotid Atherosclerosis Score
MWT: maximum wall thickness
ACS: acute coronary syndrome
MAPK: mitogen-activated protein kinase
MR-VPD: MR-vulnerable plaque diagnostics
FDA: food and drug administration
